# Sleep Strategies of Night-Shift Nurses on Days Off: Which Ones are Most Adaptive?

**DOI:** 10.3389/fneur.2014.00277

**Published:** 2014-12-19

**Authors:** Megan E. Petrov, C. Brendan Clark, Hylton E. Molzof, Russell L. Johnson, Karen L. Cropsey, Karen L. Gamble

**Affiliations:** ^1^College of Nursing and Health Innovation, Arizona State University, Phoenix, AZ, USA; ^2^Department of Psychiatry and Behavioral Neurobiology, University of Alabama at Birmingham, Birmingham, AL, USA

**Keywords:** shift work, nurses, chronotype, sleep disturbance, cardiovascular symptoms

## Abstract

**Objectives:** To determine the off-shift sleep strategies of bi-ethnic night-shift nurses, the relationship between these sleep strategies and adaptation to shift work, and identify the participant-level characteristics associated with a given sleep strategy.

**Methods:** African-American and non-Hispanic White female, night-shift nurses from an academic hospital were recruited to complete a survey on sleep–wake patterns (*n* = 213). Participants completed the standard shiftwork index and the biological clocks questionnaire to determine sleep strategies and adaptation to night-shift work. In addition, chronotype was determined quantitatively with a modified version of the Munich ChronoType Questionnaire. Most participants worked ~3 consecutive 12-h night-shifts followed by several days off.

**Results:** Five sleep strategies used on days off were identified: (a) night stay, (b) nap proxy, (c) switch sleeper, (d) no sleep, and (e) incomplete switcher. Nap proxy and no sleep types were associated with poorer adaptation to night-shift work. The switch sleeper and incomplete switcher types were identified as more adaptive strategies that were associated with less sleep disturbance, a later chronotype, and less cardiovascular problems.

**Conclusion:** Behavioral sleep strategies are related to adaptation to a typical night-shift schedule among hospital nurses. Nurses are crucial to the safety and well-being of their patients. Therefore, adoption of more adaptive sleep strategies may reduce sleep/wake dysregulation in this population, and improve cardiovascular outcomes.

## Introduction

About 15% of the United States’ workforce participates in evening, night, or rotating shift work (1). This population is at greater risk for numerous health problems including depression, cancer, gastrointestinal diseases, and cardiometabolic disorders such as obesity, heart disease, and hypertension (2–6). One of the mechanisms for these increased risks is circadian rhythm misalignment (2). Misalignment occurs when the neural biological clock that regulates daily fluctuations in sleep/wake, hormonal, and metabolic rhythms is not in synchrony with social, sleep/wake and feeding behavioral patterns, and the external environment.

Circadian misalignment is also associated with decreases in alertness and job performance. These deficits are particularly salient for night-shift, hospital nurses whose ability to regulate attention and sustain performance is vital for the safety of their patients. The traditional night-shift schedule for hospital-based nurses is that of two or three, sequential 12-h night shifts from 7:00 p.m. to 7:00 a.m. followed by two to five off-shift days. This schedule of work to non-work days is equivalent to jet lag resulting from undergoing a Tokyo–San Francisco roundtrip every few days. Previous studies have mostly focused on the adaptation of circadian phase to night-shift work (i.e., diurnal sleep quality and duration) and sleepiness during the night shift, with little focus on the associations of sleep on days off to health symptoms and functioning (7, 8). A rich literature has provided multiple investigations of the effects of simulated night-shift work on adaptation of circadian phase, sleep quality, melatonin, cortisol, and performance (9–13). However, these studies often require participants to follow a strict diurnal sleep schedule. These studies do not mimic real-world choices of night-shift nurses. The majority of night-shift nurses prefer to sleep at night during their off-shift days (14). Therefore, there is a need to assess adaptation to frequent switches between nocturnal and diurnal sleep and the method of making these switches because the quality of the method may differentially lead to circadian misalignment and likely many of the health conditions associated with shift work.

In our recently published study, self-reported maladaptation to the night-shift schedule by non-Hispanic White, hospital nurses was significantly associated with the selection of sleep strategies on off-shift days (14). These nurses completed a schedule of a typical night-shift work-week. Five sleep strategies for days off were characterized from the responses (14). Nurses adopting the least adaptive off-shift sleep strategies tended to be older and have more years of experience. They also were more likely to have an early chronotype (i.e., natural tendency to go to bed earlier). Limitations within this work were the lack of ethnic diversity in the sample, and no data were available to understand what individual health, sleep, and behavioral factors (e.g., mental health or metabolic disturbances) are associated with off-shift sleep choices. Given that there are ethnic differences in both endogenous clock rhythmicity (15) and metabolic responses to the environment, it is important to determine whether off-shift sleep strategies differ between ethnic groups. In addition, numerous physical and mental conditions, health behaviors, personality, and other socio-demographics may contribute to the likelihood of picking one off-shift sleep strategy or another (16).

The aims of the present study were to (1) replicate previously identified off-shift sleep strategies among bi-ethnic, hospital-based night-shift nurses, (2) determine which off-shift sleep strategies were associated with the least adaptation to night-shift work, (3) identify participant differences in socio-demographics, sleep disturbance, chronotype, personality, physical and mental health, and health behaviors across the off-shift sleep strategies and between the most and least adaptive strategies, and (4) clarify what set of worker-level characteristics predicted the use of each strategy. For Aim 1, we hypothesized that the five behavioral sleep strategies using the same operational definitions from our previous work (14) would be replicated. In addition, we predicted a similar distribution of behavioral sleep strategy types would be represented in the present sample, and that most nurses who work night-shift would prefer to switch to night-sleep on days off. For Aim 2, we hypothesized that participants’ adopting a strategy of more than 24 h without sleep either prior to the first night shift or just after the last night shift would be the least adapted to their night-shift schedule. This result was reported by Gamble and colleagues (14). For Aim 3, we predicted that older nurses, nurses with more years of shift-work experience, and nurses with an early chronotype would choose the less adaptive off-shift sleep strategies. We also hypothesized that the least adaptive off-shift sleep strategies would be associated with greater sleep disturbance and poor health. For Aim 3, we hypothesized that several socio-demographic, sleep, and health factors, notably race/ethnicity, would predict the use of the sleep strategies most associated with poor self-reported adaptation.

## Materials and Methods

### Sample and design

All female nurses employed full-time (>26 h/week) at the University of Alabama at Birmingham (UAB) Hospital (~2,700 on staff) were invited to participate in a survey study of their sleep behavior patterns through flyers distributed in nurse work areas at UAB Hospital and by email invitation over a 6-month period. For the present study, only nurses working the night-shift were included in the analyses. The hospital nurse population at UAB is diverse considering that only 5.4% of the national nursing workforce describes themselves as black or African-American (17). The UAB Hospital population consisted of 79.2% White individuals and 18.4% African-Americans. Questionnaires were administered using REDCap electronic data capture tools hosted at UAB (18). In total, 373 nurses completed the survey, of which 213 were night-shift nurses. These participants worked one (3.0%), two (22.0%), three (69.0%), or four or more (6.0%) consecutive night shifts. Only 14.7% of the 213 participants also participated in rotating shifts at least once every 2 weeks. The final sample consisted of 32 African-Americans (15.3%) and 177 non-Hispanic Whites (84.7%). The study was approved by the UAB Institutional Review Board.

### Measures

#### Biological clocks questionnaire

**Figure 1:**
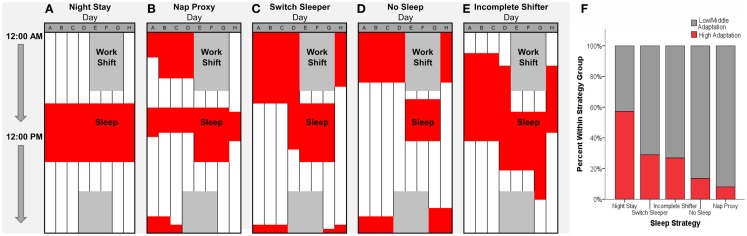
**Five sleep strategies (A–E) on off-shift days by night-shift hospital nurses (19)**. Red indicates sleep times and dark gray indicates night-shift schedule from 7:00 p.m. to 7:00 a.m. on Days D–G. **(F)** Percentage of nurses within each strategy group who indicated that their sleep patterns were well-adapted (red indicates scores of 7 or higher) to the current shift schedule. See Section “Results” for sample sizes and distribution.

The biological clocks questionnaire (BCQ) is a modified version of the Munich ChronoType Questionnaire (MCTQ, 19) by Gamble and colleagues (14). The BCQ asks participants how well-adapted they are to their current work hours with the question “On a scale from 0 to 10, how well do you feel you adapt to your current work hours?” The scale was anchored as follows: 0 indicates “not well at all, I tend to feel tired all the time, cannot enjoy my days off, and my sleep cycles never seem to be regulated”; 5 indicates “middle-of-the-road, I am okay with working this shift, but I still feel tired on my first day off, and my sleep patterns vary at times”; and 10 indicates “very well, I really enjoy this shift, have no trouble getting my energy back on my first days off, and sleep just as well when working as I do when not working.” Consistent with the prior literature (14), a cut-off value of seven indicates high adaptation. The questionnaire also contains a 7-day schedule divided into 30-min time blocks for participants to depict their current work/sleep schedules (see Figure 1). This schedule was used to define the five sleep strategy types for off-shift days according to previously determined operational definitions from Gamble and colleagues (14). The first type, night stay, is characterized by continuing to sleep regularly in the daytime on or off shift. The second type, Nap Proxy, is characterized by napping for 1 h or more for at least four out of the 5 days off during the time in which they would normally be asleep when working night-shift. The third type, switch sleeper, is characterized by switching from nights to days by sleeping late on the day they will start night-shift work or the first day off. The fourth type, no sleep, is characterized by switching from days to nights and vice versa by choosing ~24-h period to stay awake with ≤1 h of sleep in any 24-h period. The fifth type, incomplete switcher, is characterized by switching half-way between days and nights such that on at least three off-work days, they go to bed at 1:30 a.m. or later.

In addition to these sleep strategies, chronotype was determined by use of a quantitative assessment rather than asking questions related to what time of day the subject feels the best (see Composite Morningness Questionnaire below). This method has been used extensively in the MCTQ among shift workers (20, 21). This quantitative chronotype was calculated from mid-sleep time on Day B (see Figure 1), after adjustment for “sleep debt” over the entire week (21; mid-sleep free, sleep-debt corrected; MSF_SC_). “Sleep debt” was calculated according to the following equation from our previous work which modified the equation reported by Roenneberg and colleagues (22): [0.5 × (Sleep Duration on Day B − Total Sleep Duration for Whole Week)/8]. Chronotype was defined by binning MSF_SC_ into seven 1-h bins, such that the lowest bin referred to mid-sleep times that were earlier than 2:00 a.m., and the highest bin referred to times that were later than 7:00 a.m. (14).

#### Standard shiftwork index

The standard shiftwork index (SSI) is the “gold standard” for collecting information regarding the effects of shift work (23). The SSI contained socio-demographic information plus 11 sub-scales that allowed for associations to be made between defined sleep strategy types (from BCQ) and measures of (a) perceived sleep disturbance, (b) physical health questionnaire, (c) coping strategies inventory, (d) circadian type inventory (circadian rhythms characteristics other than phase such as vigorousness and flexibility), (e) job satisfaction, (f) chronic fatigue, (g) general health questionnaire (i.e., mental health status and medication use), (h) cognitive somatic anxiety questionnaire, (i) composite morningness questionnaire (subjective assessment of the subject’s “feeling best” time of day based on morning activities, morning affect and eveningness), (j) eysenck personality inventory (extraversion and neuroticism), and (k) social and domestic survey (more details can be found at http://www.workingtime.org). Within the general health questionnaire participants are asked whether they have ever used anti-depressant medications. Considering the high prevalence of depression among shift workers, this variable was also considered to be an important predictor (24).

### Analyses

For Aim 1, two independent raters scored the 7-day sleep/wake schedules for the five sleep strategy types with 90.4% agreement (Cohen’s Kappa = 0.85). Discrepancies were resolved by a third independent rater. Frequencies for each sleep strategy type were calculated as well as the proportion of participants who switched to night sleep on days off. For Aim 2, a univariate ANOVA was conducted to estimate the association between sleep strategy and self-reported adaptation to the night-shift-work schedule. For Aim 3, a series of univariate comparisons (ANOVA for continuous variables and chi-square for categorical variables) were conducted to determine sleep strategy differences among worker-level characteristics. The sleep strategies were also categorized by self-reported adaptation level according to the results of Aim 2. Then, univariate comparisons between those who adopted the less adaptive sleep strategy or strategies and those who adopted the more adaptive sleep strategies were analyzed with particular emphasis on differences in age, shift-work experience, and chronotype. For Aim 4, variables which demonstrated a significance level of 0.1 in the univariate analyses were then entered into a backward-selection, logistic regression predicting use of the most adaptive sleep strategies compared to maladaptive strategies. Analyses were performed with SPSS, Version 22 (IBM Corp., Armonk, NY, USA).

## Results

Of the 213 nurse participants (age range: 22–64 years) retained in the analyses, all were categorized exclusively into one of the five sleep strategy types. The distribution was as follows: 7 (3.3%) night stay type; 25 (11.7%) nap proxy type; 115 (54.0%) switch sleeper type; 40 (18.8%) no sleep type; and 26 (12.2%) incomplete shifter type. There were no differences in the proportion of rotating shift workers by sleep strategy type (χ^2^ = 4, *N* = 211) = 5.7, *p* = 0.22. Overall, 96.7% of participants preferred to switch to sleeping at night on their off-shift days. Only 23.9% of the sample reported high adaptation levels to their shift schedule as measured by the BCQ adaptation item with a cut-off score of seven on a scale from one to ten.

For Aim 2, there were significant differences in self-reported adaptation by sleep strategy type, *F*(4, 208) = 3.4, *p* = 0.01. Given the small sample size for Night Stay types (*n* = 7), *post hoc* analyses must be interpreted with caution. Overall, night stay types were significantly more adapted than all other types, switch sleepers were more adapted than no sleep and nap proxy types, and no different from incomplete switchers. When adaptation was stratified by the cut-off score of seven, these results were replicated (see Figure 1) (χ^2^ = 4, *N* = 209) = 11.5, *p* = 0.02. Since night stay types were so few, a univariate ANOVA was conducted to determine differences in adaptation to night-shift work among the other sleep strategy types, excluding night stay. There was a strong but not significant trend in self-reported adaptation to night-shift work by sleep strategy type, *F*(3,202) = 2.4, *p* = 0.07, see Table 1. *Post hoc* analyses indicated that switch sleeper types were no different than incomplete shifter types (*p* = 0.35), but they were significantly more well-adapted than no sleep (*p* = 0.04) and nap proxy (*p* = 0.04) types.

**Table 1:** **Univariate comparisons between sleep strategies on participant characteristics**.

Variable	Switch sleeper (*n* = 114)	No sleep (*n* = 37)	Incomplete shifter (*n* = 26)	Nap proxy (*n* = 25)	χ^2^	*F*
Age (years)	31.5 (1.0)^a^	33.9 (1.8)	32.2 (2.1)	36.8 (2.1)^a^	–	1.9
Race (*n*, %)^1^					–	–
Black	–	–	–	–		
White						
Marital status (*n*, %)					1.3	–
Married/partnered	67 (58.3)	21 (57.5)	13 (50.0)	12 (48.0)		
Single	48 (41.7)	16 (42.5)	13 (50.0)	13 (52.0)		
BMI (kg/m^2^)	26.7 (0.7)	27.6 (1.2)	27.9 (1.5)	27.7 (1.5)	–	0.3
Night-shift experience (*n*, %)					8.4*	–
<6 years	73 (64.0)	18 (45.0)	17 (65.4)	9 (39.1)		
6+ years	41 (36.0)	22 (55.0)	9 (34.6)	14 (60.9)		
Adaptation to shift	5.5 (0.2)^a,b^	4.6 (0.3)^a^	5.0 (0.4)	4.5 (0.4)^b^		2.4^†^
Chronotype	2.4 (0.1)^a^	1.9 (0.3)^b^	4.6 (0.3)^a–c^	2.3 (0.3)^c^	–	18.4**
Morningness	33.9 (0.7)^a^	33.2 (1.2)^b^	27.1 (1.6)^a–c^	34.3 (1.5)^c^	–	5.5**
Sleep disturbance	18.9 (0.4)^a,b^	20.9 (0.7)^a,d^	18.8 (0.8)^c,d^	21.1 (0.9)^b,c^	–	3.9*
Chronic fatigue	29.8 (0.8)	31.5 (1.3)	31.8 (1.6)	32.9 (1.8)	–	1.2
Neuroticism	12.6 (0.3)	13.0 (0.5)	12.8 (0.6)	13.4 (0.6)	–	0.5
Extraversion	16.2 (0.3)	16.7 (0.6)^a^	15.6 (0.7)	14.7 (0.7)^a,^	–	1.9
General health questionnaire	23.6 (0.5)^a,b^	26.6 (0.9)^a^	25.9 (1.0)^b^	25.5 (1.1)	–	4.1**
Cognitive anxiety	12.9 (0.6)^a^	15.5 (1.0)^a^	13.8 (1.2)	13.9 (1.2)	–	1.7
Somatic anxiety	14.5 (0.5)	14.8 (0.8)	14.8 (1.0)	15.0 (1.0)	–	0.1
Social and domestic activities	48.8 (1.3)	49.6 (2.2)	45.2 (2.7)	48.0 (2.7)	–	0.6
Job satisfaction	5.2 (0.1)^a^	5.1 (0.2)	5.1 (0.2)	4.6 (0.2)^a^	–	1.63
Coping engagement	50.6 (1.0)	50.4 (1.6)	50.5 (2.1)	50.2 (2.3)	–	0.01
Coping disengagement	33.7 (1.2)	37.2 (2.0)	37.5 (2.4)	35.6 (2.7)	–	1.2
Alcohol intake	1.1 (0.2)	1.4 (0.3)	1.5 (0.4)	1.8 (0.4)	–	1.0
Caffeine intake	2.7 (0.2)	3.2 (0.4)	3.0 (0.4)	3.0 (0.5)	–	0.5
Cardiovascular problems	11.0 (0.3)^a^	12.0 (0.6)	11.9 (0.7)	12.7 (0.7)^a^	–	2.3^†^
Digestive difficulties	14.8 (0.5)^a^	16.1 (0.8)	16.6 (1.0)	17.7 (1.1)^a^	–	2.6^†^
Anti-depressant medication (*n*, %)					10.8*	–
Never	87 (75.7)	24 (60.0)	21 (80.8)	12 (48.0)		
Ever	28 (24.3)	16 (40.0)	5 (19.2)	13 (52.0)		

*^1^ At least one cell had a sample size of <5*.

*Note. Values with the same superscript letters indicate that these values are significantly different from each other*.

*BMI, body mass index*.

**p < 0.01; *p < 0.05; ^†^*p* < 0.1.

Table 1 displays univariate associations between sleep strategy types with the exception of night stay, and all worker-level characteristics. For Aim 3, a univariate ANOVA indicated that there were no significant differences in age by sleep strategy type. Chi-square analyses for duration of shift-work experience by sleep strategy type resulted in a significant relationship indicating switch sleeper and incomplete shifter types had less years of experience whereas No Sleep and Nap Proxy types had more. Late chronotype was associated with Incomplete Shifter types. Morningness, sleep disturbance, mental health status, and anti-depressant medication use were also significantly differentiated across the off-shift sleep strategies. However, the small sample sizes for nap proxy and incomplete shifter types reduced power and precluded the ability to conduct a multinomial regression analysis to determine which set of worker-level characteristics predicted each sleep strategy. Therefore, strategies were categorically combined as either more adaptive (i.e., switch sleeper, and incomplete shifter, *n* = 140) or less adaptive (i.e., no sleep and nap proxy, *n* = 62). After categorization, univariate ANOVA models indicated that adaptation levels were significantly different between these two groups [*M* = 5.4, SD = 2.1 vs. *M* = 4.6, SD = 1.8, *F*(1,204) = 6.2, *p* = 0.01]. Table 2 displays univariate association between the more adaptive compared to the less adaptive off-shift sleep strategies. Regarding Aim 3, there were significant difference in age, duration of shift-work experience and chronotypes between the two groups such that participants adopting the adaptive off-shift sleep strategies were younger, had less shift-work experience and had later chronotypes.

**Table 2:** **Univariate comparisons between self-reported adaptive and maladaptive sleep strategies on participant characteristics**.

Variable	More adaptive^a^	Less adaptive^a^	χ^2^	*F*
	Switch sleeper or incomplete shifter (*n* = 140)	No sleep or nap proxy (*n* = 62)
Age (years)	31.6 (9.9)	35.1 (11.7)	–	4.5*
Race (*n*, %)			0.02	–
Black	22 (15.6)	10 (15.4)		
White	119 (84.4)	55 (84.6)		
Marital status (*n*, %)			0.2	–
Married/partnered	80 (56.7)	35 (53.8)		
Single	61 (43.3)	30 (46.2)		
BMI (kg/m^2^)	26.9 (7.3)	27.6 (8.0)	–	0.4
Night-shift experience (*n*, %)			8.2**	
<6 years	90 (64.3)	27 (42.9)		
≥6 years	50 (35.7)	36 (57.1)		
Chronotype	2.8 (1.7)	2.1 (1.5)	–	6.3*
Morningness	32.7 (7.7)	33.6 (8.0)	–	0.7
Sleep disturbance	18.8 (3.9)	21.0 (4.2)	–	11.7**
Chronic fatigue	30.2 (8.5)	32.0 (8.1)	–	2.0
Neuroticism	12.7 (3.0)	13.2 (2.8)	–	1.3
Extraversion	16.1 (3.4)	15.9 (3.6)	–	0.1
General health questionnaire	24.0 (5.0)	26.2 (6.0)	–	7.3**
Cognitive anxiety	13.1 (5.55	14.9 (7.0)	–	3.8^†^
Somatic anxiety	14.5 (5.2)	14.9 (4.8)	–	0.2
Social and domestic activities	48.2 (13.8)	49.0 (13.4)	–	0.2
Job satisfaction	5.2 (1.1)	4.9 (1.1)	–	2.0
Coping engagement	50.6 (10.0)	50.3 (8.8)	–	0.03
Coping disengagement	34.5 (11.8)	36.6 (11.7)	–	1.2
Alcohol intake	1.2 (1.8)	1.6 (2.2)	–	1.6
Caffeine intake	2.8 (2.2)	3.1 (2.4)	–	1.0
Cardiovascular problems	11.2 (2.9)	12.3 (4.2)	–	4.7*
Digestive difficulties	15.1 (5.0)	16.7 (5.5)	–	4.18*
Anti-depressant medication (*n*, %)			9.5**	–
Never	108 (76.6)	36 (55.4)		
Ever	33 (23.4)	29 (44.6)		

^†^*p* = 0.1, *p < 0.05, **p < 0.01.

*^a^Values are in means and SD unless otherwise noted*.

In addition, the adaptive group tended to have less sleep disturbance, better mental health, less cognitive anxiety, reduced cardiovascular problems, fewer digestion difficulties, and were less likely to use anti-depressant medication. There were no differences for ethnicity or any other variables of interest. However, ethnicity was significantly related to chronotype with African-American nurses reporting an earlier chronotype than non-Hispanic White nurses [*M* = 2.0, SD = 1.6 vs. *M* = 2.7, SD = 1.7, *F*(1,190) = 4.0, *p* = 0.046, $\eta_{p}^{2}=0.02$].

The results of the binary logistic regression can be seen in Table 3. The adaptive sleeping patterns were associated with a later chronotype, less sleep disturbance, and fewer cardiovascular problems. Age, digestive problems, night-shift experience, mental health status, cognitive anxiety, and anti-depressant use were not significant.

**Table 3:** **Logistic regression of self-reported predictors for adaptive sleep patterns (i.e., switch sleeper, incomplete switch sleeper) on off-shift days among night-shift hospital-based nurses: backward approach**.

Steps	Characteristic	Odds ratio	95%CI
Step 1	Age	0.97	0.92–1.03
	Chronotype	1.34	1.01–1.79
	Sleep disturbance	0.88	0.77–0.99
	Night-shift experience (Ref: <6 years)	0.76	0.23–2.56
	Cardiovascular problems	0.90	0.78–1.03
	Digestive difficulties	0.96	0.87–1.05
	Anti-depressant medication (Ref: never)	0.80	0.30–2.13
	General mental health questionnaire	1.05	0.94–1.17
	Cognitive anxiety	0.99	0.89–1.09
Final step	Chronotype	1.41	1.08–1.84
	Sleep disturbance	0.90	0.80–0.99
	Cardiovascular problems	0.87	0.77–0.98

*Final model: χ^2^ = 21.0, df = 3, *p* < 0.001*.

## Discussion

Bi-ethnic, night-shift nurses working at a large metropolitan hospital predominantly reported that they are not well-adapted to night-shift work, and that they prefer to sleep at night during their off-shift days. Depending on the sleep strategy they used to make the switches between nocturnal and diurnal sleep, their degree of adaptation to night-shift work varied considerably. Those who reported they were the least well-adapted to their work schedule (~30%) also reported that in their attempts to switch from nocturnal to diurnal sleep they either (1) took a long nap on their off-shift days (i.e., Nap Proxy) or (2) chose ~24-h period to stay awake to help them make the switch (i.e., no sleep, usually prior to their first work shift). These individuals tended to be older and have more shift-work experience, suggesting that age and experience may not lend itself to choosing the best sleep strategy. Participants reporting moderate to good adaptation to night-shift work for the most part reported taking a more graduated approach to switching from nocturnal to diurnal sleep by gradually staying up later and getting out of bed later (i.e., switch sleeper and incomplete switcher types). The characteristics of those adopting the adaptive sleep strategies on their off-shift days were later chronotype, less sleep disturbance, and fewer cardiovascular problems.

For all study aims, the results generally supported our hypotheses. Night-shift nurses prefer to sleep at night during their off-shift days. The distribution of the five sleep strategies overall were comparable to our previous work (14), with two exceptions. Less participants used the No Sleep strategy (~19 vs. 25%) while more participants used the Switch Sleeper strategy (~54 vs. 50%). Switch Sleeper type was still the most common, which is similar to data collected from other studies suggesting sleep duration is longer the night before the first night shift and often includes a daytime nap (25, 26). Identical to Gamble et al. (14), Nap Proxy and No Sleep types were also the least adapted to night-shift work. Similar to that study, these types were significantly more likely to be early chronotypes whereas participants adopting adaptive sleep strategies were more likely to be later chronotypes. The characteristics associated with more adaptive sleep strategies are comparable to those reported by a systematic review on individual differences in shift-work tolerance such that low scores on morningness (akin to the chronotype variable in the present study) were associated with greater tolerance (27). Dissimilar to the results of this review, our findings indicate that personality features of extraversion and neuroticism were not related to choice of sleep strategy. Though the review found high extraversion and low neuroticism to be associated with better shift-work tolerance. However, the constructs of tolerance and adaptation are likely to be different which may explain these discrepancies. Further, the relationship between personality and shift-work tolerance or adaptation has only been conducted in cross-sectional studies.

Disrupted sleep is one of the most common effects of shift work. Treatment for shift-work sleep disorder in particular includes prescribed sleep/wake scheduling and circadian phase shifting (28). Prescribed sleep/wake scheduling is mostly intended to counter on-the-job excessive sleepiness rather than to promote better sleep and daytime functioning on days off. Circadian phase shifting mostly requires the use of medications such as melatonin, hypnotics, and stimulants. Our findings may spur the development of a complementary treatment to these interventions that would be intended to reduce the sleep disturbance on days off. The adaptive sleep strategies identified in the present study were associated with less sleep disturbance than the maladaptive sleep strategies. Although causality cannot be determined, these results indicate that future studies ought to test whether behavioral modification of sleep/wake patterns on off-shift days may reduce the burden of sleep disturbance.

The evidence on the association between shift-work and greater prevalence and incidence of cardiovascular diseases is moderate to robust (19, 29, 30). Numerous moderators and mechanisms have been proposed to modify or explain this relationship such as circadian rhythm misalignment. However, no study to date has documented that off-shift behavioral sleep strategies may modify the strength of this association. Our study provides compelling evidence that modifying one’s sleep–wake cycle into an adaptive pattern that allows for better sleep and energy is also related to reduced odds of cardiovascular symptoms such as palpitations and irregular heartbeat, chest pain and tightness, and high blood pressure. The pathway between behavioral sleep modification and cardiovascular symptoms may be better circadian alignment. These results suggest that development of behavioral, sleep-related interventions to improve adaptability to night-shift work, and to test whether it is associated with an improved cardiovascular profile would be a fruitful endeavor.

Almost a third of the sample reported using maladaptive sleep strategies, one of which, no sleep for ~24-h period, could be associated with substantial sleep deprivation likely to inhibit on-the-job performance on par with legal levels of alcohol intoxication (31). The results of this study do not elucidate the reasons or mechanisms for why these sleep strategies were chosen over more adaptive ones, though the findings do provide evidence of the characteristics associated with such choices. These participants along with their nap proxy type counterparts were more likely to be older and with more years of shift-work experience. Intuitively, it would be expected that with more years of experience, night-shift nurses would learn over time what sleep strategies work best for them and choose accordingly. However, within this sample, this was not the case. One reason might be that older, more experienced night-shift nurses may perceive that they are “habituated” to having a disrupted sleep–wake cycle. Thus, they may not believe their choice in off-shift sleep schedules influences their adaptation and performance in their work as much as someone who is less experienced. Alternatively, with more years of circadian misalignment, the ability to adapt to the shifting schedule may deteriorate with increasing health problems, sleep fragmentation, and excessive daytime sleepiness associated with misalignment, as well as, natural changes in chronotype due to aging from late to earlier phases. Ultimately, this may be related to the inability to shift sleep schedules with more adaptive methods but instead more dramatic options are chosen such as sleep deprivation or napping to excess. As the present study was cross-sectional, this hypothesis cannot be substantiated. Future studies should prospectively examine the sleep strategies chosen by night-shift workers on their off-days from the inception of their first shift-work experience and on.

Despite known ethnic differences in endogenous clock rhythmicity, ethnicity was not a predictor of sleep strategy type in the current sample. This may be partly due to the smaller proportion of African-Americans in the sample despite the large proportion of this ethnic group relative to other nursing facilities in the nation. African-Americans have been reported to have shorter circadian clock periods than non-Hispanic Whites and greater phase shifting responses to light (15, 32). In other words, the clock runs “faster” for individuals who have a tendency to wake early in the morning. In the present study, African-Americans did tend to have an earlier chronotype (indicative of an advanced phase angle) than their non-Hispanic White counterparts, though ethnicity had no bearing on choice of sleep strategy on days off. Nonetheless, with the exception of the studies by Eastman et al. and Smith et al., no other studies have reported evidence of ethnic differences in chronotype (15, 32).

In interpreting our findings, several factors merit consideration. First, the dataset is limited to self-reported estimates of all predictors and outcomes. Although self-estimates of maladaptation to night-shift work and sleep disturbance are appropriate to the questions at hand, prospective estimates of sleep–wake schedules over a prolonged period of time using actigraphy and sleep diaries would be informative of the regularity of the chosen sleep strategy. Second, the dataset is cross-sectional, so causality cannot be determined. Third, the sample was comprised of African-American and non-Hispanic White female nurses at one hospital site which may limit generalizability. However, few prior studies have reported on the sleep and adaptation of bi-ethnic night-shift nurses. This hospital site provided that opportunity. Fourth, napping during the night shift may have occurred which could have altered the results. However, napping was not assessed. Fifth, about 15% of the sample engaged in rotating shift work which may have altered the results; however, there were equal proportion of these participants engaging in each of the sleep strategies. Lastly, the sample sizes for the maladaptive sleep strategies as well as for African-Americans were somewhat limited. Further study of these constructs in larger, even more diverse samples would be useful.

The occupational health implications of our findings are fourfold: (1) night-shift nurses could receive recommendations for sleep/wake timing and patterns during their days off for improved sleep regularity and shift-work adaptation; (2) night-shift nurses could be informed of the consequences of the No Sleep and Nap Proxy strategies; and (3) re-evaluation of how appropriate is the present, commonly used 12-h, three consecutive nights shift schedule for optimal health of the night-shift nurse. Future studies should examine the combination of planned napping during night-shift work with sleep strategy type on night-shift adaptation. Short naps during a 12-h night shift have been found to improve work performance and alertness (33, 34). Further work must also be conducted on whether sleep strategy type is also related to safety and performance measures on-the-job.

## Conflict of Interest Statement

The authors declare that the research was conducted in the absence of any commercial or financial relationships that could be construed as a potential conflict of interest.
